# Biomonitoring of Exposure to Metals in a Population Residing in an Industrial Area in Brazil: A Feasibility Study

**DOI:** 10.3390/ijerph182312455

**Published:** 2021-11-26

**Authors:** Élida Campos, Carmen Freire, Fernando Barbosa, Cristina Lemos, Valéria Saraceni, Rosalina J. Koifman, Rafael do Nascimento Pinheiro, Ilce Ferreira da Silva

**Affiliations:** 1 Department of Epidemiology and Quantitative Methods in Health, National School of Public Health, Oswaldo Cruz Foundation (FIOCRUZ), Rio de Janeiro 21040-900, Brazil; cfreire@ugr.es (C.F.); rosalina.koifman@hotmail.com (R.J.K.); ilce23@hotmail.com (I.F.d.S.); 2 Instituto de Investigación Biosanitaria de Granada (ibs. GRANADA), 18012 Granada, Spain; 3 CIBER de Epidemiología y Salud Pública (CIBERESP), 28029 Madrid, Spain; 4 School of Pharmaceutical Sciences of Ribeirão Preto, University of São Paulo (USP), São Paulo 05508-060, Brazil; fbarbosa@fcfrp.usp.br; 5 Superintendence of Health Surveillance, Municipal Health Secretariat of Rio de Janeiro, Rio de Janeiro 20211-901, Brazil; mcrislemos@hotmail.com (C.L.); valsaraceni@gmail.com (V.S.); rpinheiro.rio@gmail.com (R.d.N.P.)

**Keywords:** survey, human biomonitoring, feasibility, toxic metals, steel industry, Brazil

## Abstract

Background: Industries are sources of environmental pollutants. However, there are few human biomonitoring (HBM) studies in the vicinity of industrial areas. Thus, we evaluate the feasibility of conducting an HBM study to assess exposure to metals in an industrial area in Rio de Janeiro, Brazil. Methodology: A cross-sectional survey was conducted near a steel factory. Adults (exposed = 775; controls = 775) were randomly selected and sex-matched. Subjects were interviewed using a questionnaire and a 24 h dietary recall. Blood samples were collected to analyze metal concentrations, blood count, biochemical parameters, and thyroid hormones. The feasibility of the survey was assessed following guidelines. The descriptive analysis was performed for the first 250 participants (pilot study). Results: Adjustments were made to the survey execution, including age-matching, fieldwork team, questionnaire, blood collection, and research awareness. The complete questionnaire was answered by ≥97% of participants; metals were measured in ≥98% and clinical parameters in ≥89%, except thyroid hormones (13–44%). The average age and family income were of 50 years and USD 575/month, respectively. The participants had equal distribution among sexes: 50% had a medium education level, and 59% were nonwhite. Conclusion: This preliminary HBM study demonstrates feasibility for the total population, with results indicating representativeness of the target population.

## 1. Introduction

Industrial activities are critical anthropogenic sources of environmental pollution. Pollutants emitted by factories are heterogeneous and depend on the activities and production processes involved, such as the petrochemical industry, foundries, steel plants, metallurgy, glass, ceramics, plastics, and others [1]. Steel plants are among the most polluting industries, as their residues may contain complex organic compounds and metals, such as iron (Fe), chrome (Cr), copper (Cu), nickel (Ni), and zinc (Zn) [2]. A recent systematic review of human biomonitoring (HBM) studies in populations residing near industrial sites, carried out by our research group [3], showed higher levels of exposure to toxic metals in populations residing in the vicinity of industrial sites than in those in more distant areas. Exposure to industrial emissions may contribute to preclinical hormonal, hematological, hepatic, and renal alterations [4,5,6], and in the long term, it can increase the risk of respiratory diseases cutaneous, neurological, renal and liver conditions [7], and cancer [8].

Potentially polluting industries are frequently settled in developing countries with less strict environmental and labor legislation [9]. Thus, understanding the adverse health effects of exposure to industrial pollution has become a priority in these countries. However, unlike developed countries such as the USA [10], Germany [11,12,13], and Canada [14], in Brazil, a nationwide HBM program for surveillance of exposure to common environmental pollutants and identification of potential risk groups is lacking. Some Brazilian studies assessed exposure to toxic metals among blood donors and subjects from the general population. These studies measured blood concentrations of arsenic (As), lead (Pb), cadmium (Cd), manganese (Mn) [15,16], and Ni [17] in blood donors in the state of Acre; As, Pb, Cd, Cu, Mn, and mercury (Hg) in blood donors and young populations (children and adolescents) in São Paulo [18,19,20,21,22]; and cobalt (Co), As, Cu, Cr, Mn, Ni, Zn, Pb, and Cd in blood donors and the general population in the state of Paraná [23,24] and five other Brazilian states [25]. Few Brazilian studies investigated occupational exposure to toxic metals [26,27,28]. However, studies in populations residing near industrial areas are still scarce. Only one study assessed Mn concentrations in hair, nail, and saliva of individuals residing near (1.5 km) a refinery of ferromanganese in the state of Bahia, showing higher Mn exposure levels in subjects living more distant from the contamination source (2.5 km) [29]. The study suggests that the physical-chemical characteristics of Mn resulted in wide dispersion, with higher pollution levels in more distant areas. However, it is possible that other potential sources of Mn not taken into accounts, such as diet or traffic-related sources [30], contributed to Mn exposure, highlighting the need to consider environmental and lifestyle factors when assessing the impact of industrial emissions on the exposure of the population residing in the surrounding areas.

In our review of HBM studies on exposure to metals in industrial areas, we observed a lack of HBM studies in Latin American countries, with most studies being conducted in Europe, North America, and Asia [3]. In addition, several methodological flaws were identified, such as the absence of a comparison group and the use of non-probabilistic sampling methods [3]. These weaknesses limit the internal validity of studies as well as the generalization of results. In addition, small population samples [31], the use of individuals residing too close to the industrial area as comparison groups [32,33], and the lack of validation of metal analysis [33,34,35] hamper the ability to estimate the magnitude of exposure to industrial pollutants adequately. One way to overcome these methodological issues would be to conduct feasibility studies before carrying out the research. This type of study allows the ascertainment of whether the investigation is feasible or necessary to make adjustments to the methodology and execution of the study [36]. Therefore, it is important to assess the feasibility of research strategies to overcome barriers, such as the difficulties researchers face in accessing technologies, including specific laboratory analyses (e.g., metals analysis), and better communication and partnership among governmental and academic sectors.

Between 2010 and 2012, several severe pollution episodes caused by leaks from a steel plant pig iron storage tank occurred in the Santa Cruz industrial district [37]. After these episodes, the local population reported respiratory, eye, and dermatological problems [38]. After these episodes, environmental monitoring activities carried out in the region revealed the presence of several metallic elements, including aluminum, arsenic, barium, bromine, calcium, cadmium, cerium, chlorine, chromium, copper, iron, potassium, lanthanum, magnesium, manganese, neodymium, phosphorus, lead, praseodymium, rubidium, sulfur, strontium, silicon, and zinc, in the air in the surrounding area and the waste deposited on the roofs of houses [39]. However, no epidemiological study has assessed the exposure to the environmental pollutants originating in this factory and their potential adverse health effects among residents in this area. In this context, the present study aimed to evaluate the feasibility of an HMB survey to assess exposure to environmental metals in a population residing in an industrial area in Rio de Janeiro, Brazil.

## 2. Methods

The present study is part of a major project called “Environmental biomonitoring and assessment of exposure to metals and their adverse health effects in the population residing in the industrial district of Santa Cruz, Rio de Janeiro—RJ,” which is the result of a partnership between the National School of Public Health-FIOCRUZ (ENSP/FIOCRUZ) and the Rio de Janeiro Municipal Health Department. The ENSP/FIOCRUZ Research Ethics Committee (CEP) approved this study in August 2016 (CAAE registry number 1,677,230), and all participants signed a free and informed consent term (TCLE in the Portuguese acronym). The study’s feasibility was assessed following the guidelines proposed by Orsmond and Cohn [36], adapted to observational studies. According to these guidelines, the aspects evaluated were

(1) Recruitment capacity and characteristics of study participants;
(2) Data collection procedures and outcome measurements;
(3) Acceptability and adaptation of study procedures;
(4) Resources and ability to manage and implement the study;
(5) Preliminary evaluation of participants’ responses.

### 2.1. Human Biomonitoring Study

#### 2.1.1. Study Location

**Figure 1:**
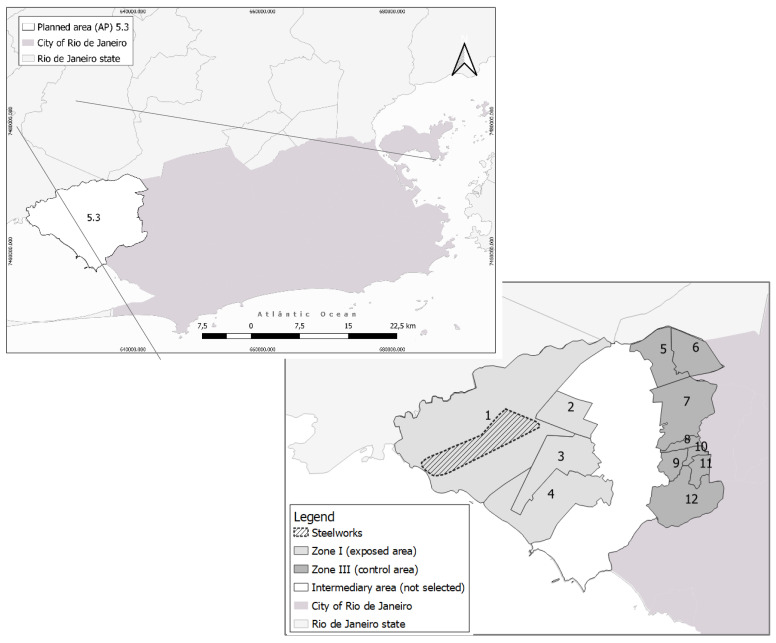
Planning area 5.3 of the city of Rio de Janeiro and the territories of the health units included in the study (zone I: 1 to 4, zone III: 5 to 12).

This study used partial data from the major project, conducted in the health planning area (AP) 5.3 in Rio de Janeiro, which includes the neighborhoods of Santa Cruz, Sepetiba, and Paciência. AP 5.3 has a surface area of 164.05 km^2^ [40] and 368,534 inhabitants [41]. The area includes the Santa Cruz industrial district, where a steel plant has been working since 2010. The factory can produce 10 million tons of steel per year, including a thermoelectrical plant and a port [37]. AP 5.3 was divided into three zones according to the health units to characterize the different scenarios of exposure for the population residing in the area (Figure 1): (I) exposed area (included in the study)—up to 8 km distant from the steel plant (the center of the plant); (II) intermediate area (not included in this study)—between 8 and 10 km distant from the steel plant; and (III) control area (included in this study)—between 10 and 14 km distant from the steel plant. Each health unit covers different micro areas, so zone I contain six micro areas distributed throughout four health units (1 to 4), whereas one III includes seven micro areas distributed throughout eight units (5 to 12).

#### 2.1.2. Study Population

The study population included adult subjects of both sexes covered by the Brazilian Family Health Strategy Program and residing zones I and III of AP 5.3 in 2017–2018. Inclusion criteria were 18 years or older and had resided in the study area for at least one year before the interview. Exclusion criteria included the inability to understand and respond to the research questionnaire due to health conditions such as cognitive impairment.

#### 2.1.3. Population Sampling and Recruitment

Participants were selected through simple random sampling based on a residents’ list of AP5.3 obtained from the Family Health Strategy Program. Sampling was performed for each zone (I and III) and distributed proportionally in the thirteen micro areas. In 2016, the adult population registered in zones I and III in the Family Health Strategy Program amounted to 69,896 and 81,715, respectively. Based on a metal exposure prevalence of 0.5, a confidence level equal to 0.95, an admissible error of 0.04, and an estimation of refusals of 0.30, the total sample estimation was 1550 participants (775 in each zone).

Half of the population sample was selected through random sampling and then matched by sex. Sex-matched participants were recruited at the same residence as those selected randomly or at the nearest address (Supplementary Material, Figures S1 and S2). Recruitment began in August 2017 in zone I. Participants received an identification code referring to the number of their zone, their micro area, and their position on the Family Health Strategy Program list. Following a Public Prosecutor’s Office (MP, in the Portuguese acronym) request, this study also included individuals with lawsuits against the steel plant.

#### 2.1.4. Research Questionnaires and Forms

The in-home interview-administered questionnaire employed in this study was based on the questionnaire used in an HBM survey carried out among blood donors in the state of Acre, Brazil [15,16]. The seven-section structured questionnaire included socioeconomic and demographic characteristics, occupational and environmental exposures, dwelling conditions, vitamin intake, gum-chewing habits, alcohol consumption, smoking habits, anthropometric information, and general health conditions. A second questionnaire, administered on the blood sampling day, was drawn to update exposure information in the past 24 h, including smoking, vitamins intake, alcohol consumption, exposure to heavy motor vehicle traffic, activities conducted (e.g., fishing, painting), and contact with specific objects (e.g., jewelry). Dietary information was collected using the Spanish version of the 24 h dietary recall Multiple-Pass Method Approach from the United States Department of Agriculture (USDA) [42]. Two of the study coauthors (I.F. and C.F) translated this dietary recall from Spanish to Brazilian Portuguese.

#### 2.1.5. Fieldwork Team Training

The fieldwork team consisted of health surveillance agents from the Health Secretary’s Office of the municipality of Rio de Janeiro. The training process of the fieldwork team included a presentation of the objectives of the study, researchers involved, data collection instruments (informed consent, questionnaires, and forms), analysis and discussion of research material, and interview simulation. All agents participated in the simulated interview as interviewers and were interviewed using the data collection material. Agents were provided with uniforms, material for field annotations, and the recruitment protocol (Figures S1 and S2) and then divided into two groups: one group interviewing participants and the other group giving support to the health units. Both teams received the same training so that staff members could change their functions according to their adaptation and study-specific needs. Managers of health units, laboratory technicians, nurses, and drivers all received the same training, except for the interview section. In addition, the nursing staff and the laboratory technicians were trained for blood collection, identification, and storage of biological samples, whereas the driver was trained for transporting biological material.

#### 2.1.6. Approach at Participants’ Residences and the Health Unit

The interviews were conducted at participants’ residences by a trained health surveillance agent who administered the questionnaire and the first 24 h dietary recall. Blood sampling was scheduled during the visit to the residence. Participants were instructed to answer the second 24 h dietary recall relative to the day before the blood sample was collected.

All participants were referred to the health unit nearest their residence to have their blood collected. Before blood drawing, subjects answered the questionnaire on recent exposures. When a participant had difficulty filling the second 24 h recall, a trained agent provided support, and the recall was completed at the health unit.

#### 2.1.7. Blood Collection and Analysis

A trained professional collected 10 mL of whole blood from each participant in the morning (between 7:00 and 8:30 a.m.) after fasting for 12 h. Samples were delivered to the laboratory on the same day blood was collected for blood count tests (red blood cells, hemoglobin, hematocrit, mean cell volume [MCV], mean cell hemoglobin [MCH], the concentration of mean cell hemoglobin [CMCH], red cell distribution width [RDW], white blood cells, neutrophils, eosinophils, basophils, lymphocytes, monocytes, and platelets), and triglycerides, total cholesterol, creatinine, urea, alkaline phosphatase, aspartate aminotransferase (AST), and alanine aminotransferase (ALT) tests. In addition, serum levels of free thyroxine (FT4), total triiodothyronine (TT3), and thyroid-stimulating hormone (TSH) were measured. Laboratory reference values (RVs) and blood analysis methods (i.e., automation and chemiluminescence) are described in Table S1.

An additional 10 mL of blood sample was collected using two free-metal Vacutainer tubes (Becton Dickinson^®^ (BD), Franklin Lakes, NJ, USA), one containing EDTA and the other without EDTA. The tubes were stored at 4 °C until delivery to the Molecular Epidemiology Laboratory at ENSP/FIOCRUZ. Approximately 2 mL of plasma and 2 mL of blood were obtained and stored at −80 °C until shipment (within 14 months) to the Faculty of Pharmaceutical Sciences Laboratory of the University of São Paulo in Ribeirão Preto for metal analysis. The metallic elements analyzed included the following nonessential elements: aluminum (27 Al), arsenic (75 As), barium (138 Ba), beryllium (9 Be), bismuth (209 Bi), cadmium (111 Cd), cesium (133 Cs), strontium (88 Sr), mercury (202 Hg), lithium (7 Li), nickel (60 Ni), lead (208 Pb), rubidium (85 Rb), and thallium (205 Tl), and the essential metals cobalt (59 Co), copper (63 Cu), magnesium (24 Mg), manganese (55 Mn), selenium (82 Se), and zinc (64 Zn). Metal concentrations were quantified in whole blood and plasma to obtain a suitable exposure biomarker for each metal (e.g., organic Hg in whole blood and inorganic Hg in plasma) [43]. Metal analysis was performed using -quadrupole inductively coupled plasma-mass spectrometry (q-ICP-MS) and dynamic reaction cell (DRC-ICP-MS) according to the methodology proposed by Batista et al. [44]. Samples were diluted in a 1:50 ratio in a 15-mL Falcon^®^ (BD) polypropylene tube with a solution containing 0.01% *v*/*v* Triton^®^ X-100, 0.2% *v*/*v* nitric acid, and 10 μg/L of each of the internal standard rhodium (Rh) and iridium (Ir). The accuracy and precision of the data were evaluated using blood and plasma reference materials by the National Institute of Public Health of Québec, Canada, and SERONORM, Norway. The agreement between the detected values and the target values was assessed by the matched *t*-test (95% confidence interval). The detected values were statistically in agreement with the target values [44].

The essential and nonessential metals analyzed were selected based on their public health relevance (i.e., As, Cd, Hg, Pb, Ni) or due to their occurrence in air or dust after the steel plant started to operate in Santa Cruz (i.e., As, Cd, Hg, Mn, Pb, Ni, Al, Cu, Mg, Zn) [39]. Al and Mg concentrations were only analyzed in plasma. The limits of detection (LODs) were previously reported [44,45,46].

#### 2.1.8. Supervision of the Fieldwork

For each micro area in AP5.3, weekly fieldwork supervision occurred at the central health unit in Santa Cruz. Participants’ identification was registered at the laboratory, and research questionnaires were revised after their completion. The fieldwork team was contacted when inconsistencies or missing information were identified. In case of missing personal data, participants were contacted via phone call to collect the information. The health units were informed whenever a problem in the blood samples occurred.

#### 2.1.9. Pilot Study and Statistical Analysis

This study includes selected information regarding the sociodemographic profile and the potential sources of exposure to environmental metals reported by participants, biochemical parameters, thyroid hormone levels, blood count, and blood metal concentrations. We analyzed data from the first 228 participants and 22 residents added to the study after a request from the MP, all recruited between August 2017 and October 2018. Participants included in this preliminary analysis (*n* = 250) resided in micro areas I (August–December 2017, *n* = 97), II (January–May 2018, *n* = 71), and III (June–October 2018, *n* = 82), distributed in the four health units of zone I (i.e., exposed group).

The descriptive analysis of the characteristics of study participants was performed using central tendency and dispersion measures for continuous variables and frequency distribution, and univariate analysis using the chi-squared test for categorical variables. Regarding blood metal concentrations, values below the LOD were substituted using LOD/2. The normality of the continuous variables was evaluated using the Kolmogorov-Smirnov test. Comparisons between groups were conducted using the one-way ANOVA or Mann-Whitney test for continuous variables and the exact Fisher test or chi-squared test for categorical variables, with a Bonferroni post hoc test. A *p*-value ≤ 0.05 was considered statistically significant. Further analyses were carried out by excluding participants added to the study after a request by the MP. Statistical analyses were performed using the Statistical Package for Social Sciences (SPSS) software for Windows, version 19.0 (SPSS Inc., Chicago, IL, USA).

## 3. Results

### 3.1. Execution and Adjustments

Table 1 presents the adversities met during the execution of the pilot study and the corrective measures adopted. First, selected residents who were not found at home after three attempts were replaced by the nearest neighbor of the same sex. Such a strategy resulted in a sample with a predominance of older individuals. Therefore, the recruitment protocol was altered to comply with the age-matching criterion (±5 years). Subjects living too close to the industry (e.g., residents located on the same street as the steel plant) were less likely to participate in the study than those who lived more distant. The main reason for these refusals was fear of interference with job opportunities at the factory. Therefore, the objectives of the research and the recruitment approach were emphasized during the training of the interviewers. The research was advertised using printed informative material (Figure S3), particularly at health units.

The first questionnaire administered to participants had some incomplete information, which indicated possible gaps in the interviewers’ training, the structure of the questionnaire, or respondents’ incomprehension. Thus, the questionnaire was restructured to improve the interview flow, organizing the questions in chronological order (e.g., asking the age at the first job before questions regarding occupational exposures). The team of interviewers was reduced to only those who felt identified with the work (*n* = 11), then they underwent retraining, and participants were contacted via phone call to gather the missing information. There were some problems with collecting and storing blood samples at the health units, such as insufficient volume or cold chain failure. To minimize these problems, fieldwork supervisors proceeded regular visits to the health units to check the conditions of the blood collection rooms (e.g., room temperature, fridge temperature) and retrained the laboratory staff when necessary. Blood drawing protocols were reviewed during these retraining sessions, and the study progress was shared with the team.

Some of the selected subjects were not found at their residence during the first visit. Two additional visits were made in these cases, and an invitation card including contact information (Figure S4) was delivered to their residence. After three missed appointments for blood collection, the subject was contacted to schedule a visit at the residence to have their blood collected by a trained professional. During the study, there was a healthcare workers’ strike in the city, and it was necessary to reallocate blood drawings to a single health unit located in an area further away from the residence of some of the participants. Although the municipal government supported the research, study funding was not affected by this crisis. This change in the health unit lasted four months and slowed down the attendance rate for blood drawing during this period.

### 3.2. Preliminary Results: A Pilot Study

Except for the questions regarding time spent outdoors at AP5.3 (missing data = 19%) and employment status (missing data = 11%), questions in the questionnaire had a high frequency of response (97–100%) (Table 2). The age of the participants ranged from 18 to 88 years (mean = 50; standard deviation [SD] = 16), with no difference between sexes. Most of the participants (59%) were brown-skinned and had a stable partner. Half of the participants had secondary education, and 41% had a household income higher than two minimum wages (USD 570 considering the average exchange rate of the U.S. dollar at Brazilian real (BRL) 3.50 in 2017–2018). Twenty-one percent of the subjects were retired, and 29% were unemployed at the time of the interview. A bus (or uber/taxi) driver was the most common job among economically active people. Most of the participants (70%) worked near highways or jam-packed roads, and less than half (43%) worked outdoors. Very few subjects reported exposure to metals in their workplaces. The average time of residence in the study area was 30 years (SD = 17). More than half of the participants reported living near a highway or busy road or spending 3 to 8 h a day outdoors in the area where they lived. Only 3% of participants used water from an alternative source other than the regularly provided by the government for drinking or cooking, and 22% consumed home-grown food. Active and passive smoking was reported by 34% and 48% of participants, respectively, whereas chewing gum and the presence of dental amalgam filling were reported by 15% and 34%, respectively. In micro area III, a higher frequency of participants reported residing near roads or sites with heavy motor vehicle traffic compared with micro area I, whereas in the latter, the frequency of active smoking and having an income > USD 570 was higher than in micro area II and micro areas II and III, respectively (Table 2).

Blood count and biochemical parameters by micro area are summarized in Table 3. Most parameters (*n* = 18) were measured in at least 95% of the participants; 10 parameters were measured in 89–93%, and only two had a high proportion of missing values (TT3: 56%; FT4: 87%). Missing values in some biochemical parameters were less likely to occur among participants from micro area II (*p* ≤ 0.05 for neutrophils, basophils, and TT3), whereas FT4 could not be measured in micro area II during the study period. Platelets, red blood cells, hemoglobin, hematocrit, MCV, RMCV, and RDW values were altered (< or >RV) in 5–24% of the sample. For leukocytes, alterations were observed mainly for neutrophils (22%) and eosinophils (28%). Alterations were also observed in urea (5%), creatinine (3%), AST (9%), ALT (10%), and alkaline phosphatase (14%). Half of the participants had optimum total cholesterol levels, and 65% had normal triglyceride levels. All FT4 and most TSH (91%) and TT3 (99%) levels were within RVs. The MCV and alkaline phosphatase levels of residents in the micro-area I were lower than residents in micro-area III, whereas monocyte values were lower in the micro-area I than in the micro-area II and III (Table 3). Differences in alkaline phosphatase and monocytes among micro-areas I and III remained statistically significant after excluding the MP’s participants.

Blood metal concentrations were measured in 98–99% of participants, with only 2 losses of whole blood samples and 4 losses of plasma samples in the micro-area I (Table 4). Toxic metals geometric mean concentrations in whole blood and plasma were 4.82 and 8.33 µg/L for As; 0.25 and 0.03 µg/L for Cd; 0.92 and 0.17 µg/L for Hg; 3.21 and 1.51 µg/L for Ni; and 20.8 and 1.46 µg/L for Pb, respectively. The mean concentration of Al in plasma was 24.2 µg/L; the mean concentrations of Mn, Cu, and Zn in whole blood were 21.1, 878, and 3960 µg/L, respectively, whereas in plasma, the mean concentrations were 3.55 µg/L for Mn, 969 µg/L for Cu, 1022 µg/L for Zn, and 14,216 µg/L for Mg. Statistically significant differences were observed in the concentrations of all metals between micro-areas, except for Cu and Hg in whole blood and Hg in plasma (Table 4). When the MP’s participants were excluded, the difference in blood As levels between micro-areas II and III did not remain. In contrast, statistically significant differences were observed in the plasmatic concentration of Ni between micro-area I and II and in plasmatic Al and Cd concentrations between micro-area II and III.

## 4. Discussion

The current study shows that HBM surveys are feasible and can be conducted in Brazil through a partnership between a research institution and health services. Other developing countries with similar organizational structures might use the suggested model to make large-scale cross-sectional studies possible. The adjustments made (including retraining, reduction in staff members, and changes in the questionnaire) improved the quality of data collection, which increased by up to 17%, with no significant increase in nonresponse rate after adjustments were made. However, information on the workplace was incomplete for most of the participants, hampering the identification of all subjects working in the steel plant.

The comparison of the results of this pilot study with the data of the last official census available per neighborhood showed a similar distribution by sex and education level (males: 47 vs. 46%; literate: 96 vs. 97%) [41]. However, a difference was observed between the study sample and the population base regarding skin color, age, and income, even after excluding the individuals included by the MP. In this study, the proportion of nonwhites was higher, and that of whites was lower than in the base population (nonwhites: 49 vs. 59%, whites: 34 vs. 26%) [41]. The results of the National Household Survey (PNAD, in the Portuguese acronym) suggest that the Brazilian population has changed its self-perception of skin color over the last decade [47], which might explain the difference observed. For the household income, the median value in this study was approximately 1.6 times higher than that reported in the last census (USD 291 vs. USD 457) [41].

Besides the time interval between the last census and this study, the beginning of the activities of the local steel plant in 2010 may have contributed to increased job opportunities and, consequently, to the increase in the average income of the population residing in the study area. Another possible explanation is the fact that the area of the pilot study (zone I) does not include a critical region of marginalized areas (“favelas”) in the neighborhood, as the population in the “favelas” has a lower income than other parts of the city [48]. Regarding age distribution, the study sample presents approximately 24% fewer young individuals (18–34 years old) and a higher proportion (12% higher) of elderly individuals, especially those between 60 and 69 years old, compared with the base population [41]. As questionnaires were administered during weekday business hours, older and retired people were more likely to be found at home, indicating a need to extend recruitment to weekends and holidays. This hypothesis was corroborated by the higher frequency of losses and refusals among subjects 18–59 years old (87%) compared with individuals participating in the study (data not presented). However, after matching subjects for age, the distribution of age between adult and elderly subjects was more similar to that of the base population (before matching, 18–59 years old: 86 vs. 62%, ≥60 years old: 14 vs. 39%; after matching, 18–59 years old: 86 vs. 73%, ≥60 years old: 14 vs. 28%) (IBGE, 2010). Nonetheless, comparisons with the census are limited by the time interval of 7–8 years between our study and the census, excluding subjects with cognitive impairment and data collection only in zone I of the study area.

The need for special authorization by the municipal government to perform thyroid hormone measurements suggests that administrative problems may reason for the high number of missing values for these parameters. Part of the health management in AP5.3 occurs at the unit to which blood collection was transferred during the strike (micro area II), which might present a different working scheme than other units, resulting in a reduced number of missing values for some parameters in this period. Although FT4 was not measured in micro-area II, total T4 was measured in 96% of the participants during the strike (data not shown), suggesting an error in the request of this parameter. Hematological, lipid, renal, and hepatic parameters of participants in this pilot study were similar to the profile reported for adults of the same age and sex (men between 18 and 30 years old) in Pelotas, state of Rio Grande do Sul (Southern Brazil), except for lower mean values for CHCM (34 vs. 33 g/dL) and urea (39 vs. 29 mg/dL) and higher for basophils (0.03 vs. 0.06 103/uL) in Santa Cruz [49]. In São Paulo (Southeastern Brazil), the levels of total cholesterol, triglycerides, and urea in young adults were also similar to those in participants under 30 years old in this study, whereas creatinine was higher for smokers in Santa Cruz (0.6 vs. 0.8 mg/dL) [50]. Among the hematological parameters, only eosinophils (nonsmoker/smoker, São Paulo: 0.3/0.2 vs. Santa Cruz: 0.1/0.4 103/µL) and hemoglobin (nonsmokers: SP: 15 vs. Santa Cruz: 14 g/dL) were different between present participants and those in São Paulo [50]. In the Brazilian Longitudinal Study on Adult Health (ELSA-Brazil) baseline population, the median triglyceride level was 112 mg/dL, and the mean total cholesterol level was 215 mg/dL [51]. Median levels of ALT (22 U/L) and AST (23 U/L) in individuals without nonalcoholic fatty liver disease in that same study were close to those in the present study [52]. Considering the euthyroid population (TSH = 0.4–4.0 µUI/mL), the mean level of TSH in ELSA-Brazil was lower than that in Santa Cruz (1.7 vs. 1.9 µUI/mL) [53].

Brazilian studies that assessed exposure to environmental metals used mainly whole blood as the biological matrix [15,16,17,19,20,21,22,25]. Although plasma may be suitable for metal exposure assessment, this matrix is not frequently used in HBM studies [43], hampering the comparison of metal concentrations in plasma found in this study. For nonessential metals (Table 5), the mean blood concentrations of Cd and Pb in Santa Cruz were similar to those of adults from the general population in São Paulo (Cd: 0.2; Pb: 19 µg/L) [19]. However, Cd was higher and Pb lower compared with concentrations found in blood donors from the state of Acre (Cd: 0.1; Pb: 37 µg/L) [15,16] and São Paulo (Cd: 0.1; Pb: 24 µg/L) [20,22]. The mean concentrations of As (4 µg/L) and Ni (0.7 µg/L) in these blood donors were lower than those in the present study [15,17,22], whereas for Hg, the mean level was higher (1 µg/L) [20]. Individuals from five other Brazilian states, including São Paulo, Minas Gerais, Goiás, Pará, and the Rio Grande do Sul, had lower levels of As (1.1 µg/L) and Ni (2.1 µg/L) than those in our study population, whereas they had higher Pb (65 µg/L) and Cd (0.4 µg/L) levels [25]. International studies reported lower blood concentrations of Ni, As, and Pb than those found in this study, whereas varied results were reported for Cd and Hg [10,14,54,55]. For essential metals (Table 5), the mean concentration of Mn in blood donors from São Paulo (13 µg/L) was lower than that in this study, but the mean blood Cu level (999 µg/L) was higher [22]. Similarly, concentrations of blood Mn were lower in adults from the states of Paraná (12 µg/L) [23], São Paulo, Rio Grande do Sul, Minas Gerais, Pará, and Goiás (9.6 µg/L) [25] but were also higher for Cu (890 µg/L) [25]. When considering populations from other countries, blood Mn concentrations were lower than those in Santa Cruz, whereas the mean Zn level was higher [10,14,54,55]. However, international data on blood Cu concentrations are lacking [10,14,54,55]. 

Regarding the population living in the vicinity of industrial sites (Table 5), a study involving subjects residing near different industries and mining activities in Tunisia observed mean blood concentrations of 29 µg/L for Ni, 0.9 µg/L for Cd, and 1.6 µg/L for As [56], which are higher (Ni, Cd) and lower (As), respectively, than those of this study. In Italy, industrial and port area residents showed a mean blood Pb concentration of 0.02 µg/L [57], which is lower than our mean Pb value. In other studies carried out near industries in Europe and Asia, blood concentrations of Cd [58,59,60,61] and Pb [34,58,59,60,61,62,63] were higher than those in Santa Cruz. For essential metals, subjects residing near areas with different industries in Italy showed lower blood concentrations of Mn (12 µg/L) and higher concentrations of Cu (971 µg/L) [60] than ours. However, these comparisons should be interpreted with caution, given that these studies present significant methodological limitations such as nonprobability sampling and the absence of a comparison group [3]. As the three micro areas under study are zone I (exposed population), differences in metal levels and clinical parameters may be related to differences in the characteristics of residents between micro areas, such as smoking habits or living near roads or highways, which will be explored in this HBM project.

The present study has some limitations, including a high number of missing values for thyroid hormones. However, as thyroid dysfunction first responds with changes in TSH levels, this parameter is recommended to assess altered thyroid function [64]. Another limitation was the difficulty in characterizing occupational exposures at the local steel plant. However, the delicate relationship between the local population’s demand and the job opportunities provided by the factory, including a question in the questionnaire regarding steel company employment, might have led to refusals during or after the interview. Therefore, shuffling questions regarding current or former occupational activities in the steel plant and occupational activities in other industrial areas could mitigate such a limitation. The lack of data on Cr exposure levels can be considered another limitation of the study. However, urine is the most suitable matrix to assess Cr exposure, especially for the trivalent form [43]. In addition, data on blood concentrations of Mg and Al were not available in our study population.

HBM studies in populations near industrial areas are scarce in developing countries [3], probably due to the lack of financial and technical resources and infrastructures required to conduct this type of survey. Therefore, this valuable study provides an approach to research in partnership with municipal health services to feasibly and efficiently develop extensive surveys in developing countries.

## 5. Conclusions

Despite the difficulties found at the beginning of the study, adjustments to its methodology and conduction of the fieldwork indicated that it is feasible to carry out an HBM survey in the total population of Santa Cruz, Rio de Janeiro. Although data in this study show differences in skin color and income of the study population relative to the official census, changes that have occurred in the population since the last census, as well as restrictions in zone I, suggest that the study population is representative of the base population but highlights the need to extend data collection to include non-business days. The preliminary assessment of metal exposure in Santa Cruz suggests higher exposure to As and Ni and lower exposure to Hg compared with blood donors and the general population of Brazil and other countries, whereas data on Cd and Pb exposures are inconsistent. However, Cd and Pb levels appeared to be lower than populations residing near industries in other countries. Regarding essential metals, blood levels of Mn were generally higher, whereas Cu and Zn levels were lower than those reported for other populations.

Considering the lack of HBM data in populations residing near industrial areas, especially in Brazil and other Latin American countries, this survey contributes not only to understanding the health status and the magnitude of human exposure to potentially toxic metals in the industrial district of Santa Cruz, but also to generating knowledge for populations in developing countries in similar situations of vulnerability.

## Figures and Tables

**Table 1:** Assessment of survey conduction and development.

Adversities	Adjustments
➢ Recruitment and characteristics of the sample	
- Sample composed mostly by older people.	- Matching by age.
- High number of refusals in areas closest to the steel plant.	- Retraining of interviewers.
	- Clarification of the project objectives in the recruitment process.
	- Informative posters at the health units.
➢ Procedure of data collection and measurement of biological parameters	
- Missing data in the responses.	- Retraining of interviewers, laboratory and biological sample transportation team.
- Difficulty recovering “missing” subjects.	- Limiting the team of interviewers to those who identified themselves with the work.
- Deterioration of the relationship with the participants.	- Editing and reorganizing the interview questionnaire.
- Clotted blood samples.	- Elaboration and printing blood collection protocols for the laboratory teams.
- Strained relationship with health unit managers.	- Regular visits to health units to evaluate the conditions of the blood collection rooms and sample storage areas.
	- Meeting of the central level of the Municipality Health Secretary’s Office with the managers of the health units.
➢ Acceptability and adaptation of study procedures	
- Population’s fear of losing services and jobs provided by the local industries.	- More promotion of the survey in the region.
- Need for covering bigger distances to collect blood during the strike of the municipality’s healthcare workers.	- Handing out invitation to participate in the study at home (in cases when the subject was not found home on the days and times of the home visit).
- Failure to show up on the scheduled days for blood collection.	- Blood collection at home.
- Difficulty locating the selected participants (absent when the house call was made).	
➢ Resources and ability to manage and implement the study	
- Strike of part of the municipality’s healthcare workers.	- Temporary reallocation of blood collection to a single unit whose staff was not affected.

**Table 2:** Sociodemographic characteristics and potential sources of exposure to metals in the study population (*n* = 250).

Variables	Total *n* (%)	Micro Area I *n* (%)	Micro Area II *n* (%)	Micro Area III *n* (%)
Sex				
Male	114 (45.6)	45 (46.4)	32 (45.1)	37 (45.1)
Female	136 (54.4)	52 (53.6)	39 (54.9)	45 (54.9)
Age				
18–59 years	170 (68.3)	59 (61.5)	52 (73.2)	59 (72.0)
≥60 years	79 (31.7)	37 (38.5)	19 (26.8)	23 (28.0)
Skin color				
Black	36 (14.5)	15 (15.5)	12 (16.9)	9 (11.1)
Brown	146 (58.6)	55 (56.7)	44 (62.0)	47 (58.0)
White	65 (26.1)	25 (25.8)	15 (21.1)	25 (30.9)
Yellow	2 (0.8)	2 (2.1)	0 (0.0)	0 (0.0)
Marital status				
Married/Living together	148 (59.2)	61 (62.9)	42 (52.9)	45 (54.9)
Divorced	22 (8.8)	7 (7.2)	5 (7.0)	10 (12.2)
Widower	23 (9.2)	13 (13.4)	4 (5.6)	6 (7.3)
Single	57 (22.8)	16 (16.5)	20 (28.2)	21 (25.6)
Education level				
University	20 (8.1)	10 (10.5)	5 (7.0)	5 (6.2)
High school	123 (49.8)	49 (51.6)	26 (36.6)	48 (59.6)
Elementary school	96 (38.9)	34 (35.8)	38 (53.5)	24 (29.6)
Illiterate	8 (3.2)	2 (2.1)	2 (2.8)	4 (4.9)
Household income				
<USD 285	66 (26.7)	21 (21.6)	21 (30.9)	24 (29.3)
USD 285–570	81 (32.8)	24 (24.7)	26 (38.2)	31 (37.8)
>USD 570	100 (40.5)	52 (53.6) ^a^	21 (30.9) ^b^	27 (32.9) ^b^
Income *per capita*				
up to USD 285	187 (77.3)	71 (76.3)	54 (80.6)	62 (75.6)
USD 285.01–570	38 (15.7)	13 (14.0)	8 (11.9)	17 (20.7)
>USD 570	17 (7.0)	9 (9.7)	5 (7.5)	3 (3.7)
Years residing in AP5.3				
1–10	26 (10.4)	13 (13.5)	7 (9.9)	6 (7.3)
11–30	102 (41.0)	37 (38.5)	32 (41.5)	33 (40.2)
>30	121 (48.6)	46 (47.9)	32 (45.1)	43 (52.4)
Busy road/s near the residence				
No	85 (34.4)	42 (44.2) ^a^	22 (31.0) ^a,b^	21 (25.9) ^b^
Yes	162 (65.6)	53 (55.8) ^a^	49 (69.0) ^a,b^	60 (74.1) ^b^
Time spent outdoors near the industrial district of Santa Cruz				
<3 h/day	41 (20.2)	15 (21.7)	11 (18.3)	15 (20.3)
3–8 h/day	117 (57.6)	41 (59.4)	32 (53.3)	44 (59.5)
>8 h/day	45 (22.2)	13 (18.8)	17 (28.3)	15 (20.3)
Water used to drink and cook				
Bottled water	5 (2.0)	0 (0.0)	1 (1.4)	4 (4.9)
Tap water	242 (96.8)	96 (99.0)	69 (97.2)	77 (93.9)
Reservoir	1 (0.4)	1 (1.0)	0 (0.0)	0 (0.0)
Other	2 (0.8)	0 (0.0)	1 (1.4)	1 (1.2)
Consumption of home-grown food				
No	194 (78.5)	79 (81.4)	59 (83.1)	56 (68.3)
Yes	53 (21.5)	16 (16.5)	11 (15.5)	26 (31.7)
Irrigation water used for home-grown vegetables				
Tap water	30 (93.8)	13 (86.7)	7 (100)	10 (100)
Well water	2 (6.3)	2 (13.3)	0 (0.0)	0 (0.0)
Smokes or has smoked cigarettes or cigars for at least 1 year				
Never smoked	166 (66.4)	61 (62.9)	50 (70.4)	55 (67.1)
Yes, currently smokes	30 (12.0)	18 (18.6) ^a^	4 (5.6) ^b^	8 (9.8) ^a,b^
Yes, smoked in the past	54 (21.6)	18 (18.6)	17 (23.9)	19 (23.2)
Has been married to/has lived with a smoker				
No	121 (72.9)	43 (70.5)	39 (78.0)	39 (70.9)
Yes	45 (27.1)	18 (29.5)	11 (22.0)	16 (29.1)
Works or has worked indoors with smokers				
No	134 (79.3)	49 (81.7)	43 (86.0)	42 (80.8)
Yes	35 (20.7)	11 (18.3)	7 (14.0)	10 (19.2)
Chews gum on a regular basis				
No	213 (85.2)	84 (86.6)	57 (80.3)	72 (87.8)
Yes	37 (14.8)	13 (13.4)	14 (19.7)	10 (12.2)
Has any dental amalgam filling				
No	162 (66.4)	62 (64.6)	51 (76.1)	49 (60.5)
Yes	82 (33.6)	34 (35.4)	16 (23.9)	32 (39.5)
Work status				
Formal job with contract	31 (12.7)	9 (9.6)	13 (18.6)	9 (11.1)
Job without contract	6 (2.4)	1 (1.1)	2 (2.9)	3 (3.7)
Self-employed	32 (13.1)	12 (12.8)	12 (17.1)	8 (9.9)
Retired	51 (20.8)	23 (24.5)	12 (17.1)	16 (19.8)
Unemployed	72 (29.4)	23 (24.5)	20 (28.6)	29 (35.8)
Other	53 (21.6)	26 (27.7)	11 (15.7)	16 (19.8)
Works outdoors				
No	50 (57.5)	20 (58.8)	18 (60.0)	12 (52.2)
Yes	37 (42.5)	14 (41.2)	12 (40.0)	11 (47.8)
Time spent working outdoors				
<30%	4 (18.11)	3 (25.0)	0 (0.0)	1 (9.1)
31–50%	12 (35.6)	1 (8.3)	6 (54.5)	5 (45.5)
>50%	18 (52.9)	8 (66.7)	5 (45.5)	5 (45.5)
Busy road near the workplace				
No	21 (25.6)	11 (34.4)	7 (25.5)	3 (13.6)
Yes	61 (74.4)	21 (65.6)	21 (75.0)	19 (86.4)
Works or has worked in/with:				
Paint factory				
No	235 (96.3)	90 (94.7)	65 (95.6)	80 (98.8)
Yes	9 (3.7)	5 (5.3)	3 (4.4)	1 (1.2)
Plastic factory				
No	237 (96.0)	94 (97.9)	67 (95.7)	76 (93.8)
Yes	10 (4.0)	2 (2.1)	3 (4.3)	5 (6.2)
Glass factory				
No	245 (99.2)	95 (99.0)	70 (100)	80 (98.8)
Yes	2 (0.8)	1 (1.0)	0 (0.0)	1 (1.2)
Gas station				
No	236 (95.5)	93 (96.9)	67 (95.7)	76 (93.8)
Yes	11 (4.5)	3 (3.1)	3 (4.3)	5 (6.2)
Electroplating				
No	232 (94.3)	87 (90.6)	67 (95.7)	78 (97.5)
Yes	14 (5.7)	9 (9.4)	3 (4.3)	2 (2.5)
Prosthetics				
No	245 (99.6)	95 (99.0)	70 (100)	80 (100)
Yes	1 (0.4)	1 (1.0)	0 (0.0)	0 (0.0)
Mining activities				
No	248 (100)	97 (100)	70 (100)	81 (100)
Yes	0 (0.0)	0 (0.0)	0 (0.0)	0 (0.0)
Ceramics				
No	236 (94.8)	94 (96.9)	67 (94.4)	75 (92.6)
Yes	13 (5.2)	3 (3.1)	4 (5.6)	6 (7.4)
Fixing batteries				
No	248 (99.2)	97 (100)	70 (98.6)	81 (98.8)
Yes	2 (0.8)	0 (0.0)	1 (1.4)	1 (1.2)
Developing photographs				
No	248 (99.2)	95 (97.9)	71 (100)	82 (100)
Yes	2 (0.8)	2 (2.1)	0 (0.0)	0 (0.0)
Metal welding				
No	241 (96.4)	93 (95.9)	69 (97.2)	79 (96.3)
Yes	9 (3.6)	4 (4.1)	2 (2.8)	3 (3.7)
Fertilizers				
No	239 (95.6)	92 (94.8)	65 (91.5)	82 (100)
Yes	11 (4.4)	5 (5.2)	6 (8.5)	0 (0.0)
Agriculture and/or livestock farming				
No	237 (94.8)	89 (91.8)	68 (95.8)	80 (97.6)
Yes	13 (5.2)	8 (8.2)	3 (4.2)	2 (2.4)
Pesticides				
No	246 (98.4)	94 (96.9)	71 (100)	81 (98.8)
Yes	4 (1.6)	3 (3.1)	0 (0.0)	1 (1.2)
Firearms				
No	232 (92.8)	88 (90.7)	69 (97.2)	75 (91.5)
Yes	18 (7.2)	9 (9.3)	2 (2.8)	7 (8.5)
Fishing activities				
No	246 (98.4)	94 (96.9)	70 (98.6)	82 (100)
Yes	4 (1.6)	3 (3.1)	1 (1.4)	0 (0.0)

AP5.3: Planning area 5.3 that includes the neighborhoods of Santa Cruz, Paciência and Sepetiba. Superscript letters in percentages indicate significant differences (*p*-value ≤ 0.05) in the frequencies of variables between the micro areas (a ≠ b).

**Table 3:** Levels and percentage of alterations for hematological and biochemical parameters (*n* = 250).

Parameters	N	Total	Micro Areas						Parameters	N	Total	Micro Areas					
			N	I	N	II	N	III				N	I	N	II	N	III
Red blood cells (10^6^/µL)	239	4.6 (0.6)	93	4.6 (0.6)	70	4.5 (0.5)	76	4.5 (0.6)	Lymphocytes (%)	231	35.2 (8.6)	92	35.5 (9.2)	68	35.8 (8.3)	71	34.2 (8.1)
<RV	35	14.6	10	10.8	16	29.9	9	11.8	<RV	9	3.9	12	4.3	3	4.4	2	2.8
>RV	1	0.4	0	0.0	0	0.0	1	1.3	>RV	171	74.0	65	70.7	53	77.9	53	74.6
Hemoglobin (g/dL)	244	13.7 (1.4)	93	13.8 (1.4)	70	13.6 (1.4)	81	13.8 (1.5)	Lymphocytes (10^3^/µL)	231	2.28 (0.6)	92	2.3 (0.6)	68	2.4 (0.6)	71	2.1 (0.6)
<RV	25	10.2	10	10.8	9	12.9	6	7.4	<RV	1	0.4	0	0.0	0	0.0	1	1.4
>RV	2	0.8	1	1.1	0	0.0	1	1.2	>RV	33	14.3	16	17.4	11	16.2	6	8.5
Hematocrit (%)	244	41.0 (4.8)	93	41.4 (4.3)	70	40.6 (3.9)	81	41.0 (5.8)	Monocytes (%)	231	6.9 (5.5–8.5)	92	7.2 (5.9–9.4) ^a^	68	6.7 (5.3–8.0) ^b^	71	6.5 (4.4–8.4) ^b^
<RV	27	11.1	9	9.7	8	11.4	10	12.3	<RV	2	0.9	1	1.1	1	1.5	0	0.0
>RV	4	1.6	2	2.2	0	0.0	2	2.5	>RV	14	6.1	11	12.0	0	0.0	3	6.1
MCV (fL)	244	89.7 (6.0)	93	89.0 (6.4)	70	90.6 (5.3)	81	89.8 (6.2)	Monocytes (10^3^/µL)	231	0.5 (0.2)	92	0.5 (0.2)	68	0.5 (0.2)	71	0.4 (0.2)
<RV	12	4.9	7	7.5	2	2.9	3	3.7	<RV	1	0.4	0	0.0	0	0.0	1	1.4
>RV	10	4.1	4	4.3	2	2.9	4	4.9	>RV	9	3.9	3	3.3	3	4.4	3	4.2
MCH (pg)	244	30.1 (2.3)	93	29.6 (2.4) ^a^	70	30.4 (2.0) ^a,b^	81	30.2 (2.4) ^b^	Platelets (10^3^/µL)	244	243 (60.6)	93	250 (62.3)	70	246 (63.2)	81	234 (55.6)
<RV	19	7.8	9	9.7	6	8.6	4	4.9	<RV	12	4.9	5	5.4	4	5.7	3	3.7
>RV	41	16.8	12	12.9	14	20.0	15	18.5	>RV	0	0.0	0	0.0	0	0.0	0	0.0
ACHC (g/dL)	244	33.5 (1.1)	93	33.2 (1.1)	70	33.6 (1.0)	81	33.6 (1.0)	Urea (mg/dL)	241	30.0 (26.0–36.0)	92	32.0 (27.0–38.0)	70	28.5 (26.0–34.3)	79	31.0 (25.0–36.0)
<RV	20	8.2	12	12.9	4	5.7	4	4.9	<RV	1	0.4	0	0.0	1	1.4	0	0.0
>RV	0	-	-	-	-	-	-	-	>RV	11	4.6	5	5.4	2	2.9	4	5.1
RDW (%)	244	13.5 (1.4)	93	13.6 (1.5)	70	13.7 (1.4)	81	13.4 (1.4)	Creatinine (mg/dL)	244	0.8 (0.7–0.9)	94	0.8 (0.7–0.9)	70	0.8 (0.7–0.9)	80	0.8 (0.7–1.0)
>RV	30	12.3	15	16.1	8	11.4	7	8.6	<RV	5	2.0	1	1.1	2	2.9	2	2.5
									>RV	2	0.8	1	1.1	1	1.4	0	0.0
White blood cells (%)	244	100 (100–100)	93	100 (100–100)	70	100 (100–100)	81	100 (100–100)	AST (U/L)	239	22.0 (19.0–27.0)	90	21.5 (18.0–27.0)	70	21.0 (18.8–25.0)	79	24.0 (10.0–29.0)
<RV	0	-	-	-	-	-	-	-	<RV	8	3.3	3	3.3	3	4.3	2	2.5
									>RV	13	5.4	4	4.4	2	2.9	7	8.9
White blood cells (10^3^/µL)	237	6.7 (2.1)	93	6.9 (2.3)	70	6.8 (1.9)	74	6.6 (2.1)	ALT (U/L)	239	20.0 (14.0–28.0)	90	18.0 (14.0–28.0)	70	19.5 (13.8–26.3)	79	21.0 (16.0–28.0)
<RV	7	3.0	4	4.3	0	0.0	3	4.1	<RV	4	1.7	2	2.2	1	1.4	1	1.3
>RV	7	3.0	3	3.2	1	1.4	3	4.1	>RV	19	7.9	5	5.6	8	11.4	6	7.6
Neutrophils (%)	224	54.1 (9.7)	92	53.1 (10.2)	68	53.7 (9.5)	64	55.9 (9.2)	Alkaline phosphatase (U/L)	243	76.0 (65.0–88.0)	94	72.0 (60.0–84.0) ^a^	70	74.5 (66.8–84.5) ^a,b^	79	81.0 (68.0–93.0) ^b^
<RV	71	31.7	30	32.6	26	38.2	15	23.4	<RV	1	0.4	1	1.1	0	0.0	0	0.0
>RV	10	4.5	3	3.3	3	4.4	4	6.3	>RV	33	13.6	13	13.8	9	12.9	11	13.9
Neutrophils (10^3^/µL)	224	3.6 (2.7–4.5)	92	3.7 (2.7–4.6)	68	3.4 (2.7–4.4)	64	3.5 (2.7–4.4)	Total cholesterol (mg/dL)	246	203 (44.6)	95	207 (45.3)	70	203 (39.7)	81	196 (47.4)
<RV	39	17.4	15	16.3	11	16.2	13	20.3	Borderline	76	30.9	26	27.4	27	38.6	23	28.4
>RV	11	4.9	4	4.3	4	5.9	3	4.7	High	45	18.3	20	21.1	12	17.1	13	16.0
Eosinophils (%)	230	2.2 (1.5–3.2)	92	2.4 (1.6–3.7)	68	2.4 (1.7–3.5)	70	2.0 (1.4–2.7)	Triglycerides (mg/dL)	245	121 (85.0–170)	94	115 (85.0–167)	70	128 (88.8–175)	81	123 (80.0–173)
<RV	14	6.1	7	7.6	4	5.9	3	4.3	Borderline	37	15.1	17	18.1	7	10.0	13	16.0
>RV	12	5.2	8	8.7	1	1.5	3	4.3	High	44	18.0	14	14.9	16	22.9	14	17.3
									Very high	4	1.6	2	2.1	0	0.0	2	2.5
Eosinophils (10^3^/µL)	230	0.2 (0.1–0.2)	92	0.2 (0.1–0.2)	68	0.2 (0.1–0.2)	70	0.1 (0.1–0.2)	TSH (µUI/mL)	242	1.9 (1.3–2.9)	93	1.8 (1.2–2.6)	70	1.8 (1.2–3.0)	79	2.1 (1.5–2.9)
<RV	52	22.6	16	17.4	12	17.6	24	34.3	<RV	4	1.7	1	1.1	1	1.4	2	2.5
>RV	12	5.2	5	5.4	1	1.5	6	8.6	>RV	18	7.4	6	6.5	6	8.6	6	7.6
Basophils (%)	223	0.6 (0.4–1.0)	91	0.6 (0.4–1.0)	67	0.7 (0.5–1.0)	65	0.7 (0.5–1.0)	TT3 (ng/dL)	110	114 (21.1)	12	117 (25.0)	68	115 (21.5)	30	111 (18.7)
>RV	2	0.9	1	1.1	1	1.5	0	0.0	<RV	1	0.9	0	0.0	1	1.5	0	0.0
									>RV	0	0.0	0	0.0	0	0.0	0	0.0
Basophils (10^3^/µL)	224	0.04 (0.03–0.06)	91	0.04 (0.03–0.06)	68	0.04 (0.03–0.06)	65	0.04 (0.03–0.06)	FT4 (ng/dL)	33	1.1 (0.1)	13	1.1 (0.2)	0	-	20	1.1 (0.1)
>RV	1	0.4	1	1.1	0	0.0	0	0.0	<RV	0	0.0	0	0.0	0	0.0	0	0.0
									>RV	0	0.0	0	0.0	0	0.0	0	0.0

RV: Reference value (reference values are provided in Table S1). For parameters following a normal distribution, the arithmetic average (standard deviation [SD]) is presented; for parameters not normally distributed, the median (25th percentile–95th percentile) is presented. MCV: mean corpuscular volume; MCH: mean corpuscular hemoglobin; ACHC: average corpuscular hemoglobin concentration; RDW: red cell distribution width; AST: aminotransferase aspartate; ALT: aminotransferase alanine; TSH: thyroid-stimulating hormone; TT3: total triiodothyronine; FT4: free thyroxine. Superscript letters on the mean (SD) or median (25–95th percentiles) indicate significant differences (*p*-value ≤ 0.05) in values/levels between micro areas (a ≠ b).

**Table 4:** Concentrations of metals in whole blood and plasma (µg/L) of study participants.

**% > LD**		**GM**	**GSD**	**MIN**	**MAX**	**P25**	**P50**	**P75**	**GM**	**GSD**	**P25**	**P50**	**P75**	**GM**	**GSD**	**P25**	**P50**	**P75**	**GM**	**GSD**	**P25**	**P50**	**P75**
**Blood**		**Total (*n* = 248)**							**Micro Area I (*n* = 95)**					**Micro Area II (*n* = 71)**					**Micro Area III (*n* = 82)**				
As	100	4.82	1.21	3.35	13.3	4.75	4.75	5.21	5.01 ^a^	1.19	4.52	5.02	5.38	4.82 ^b^	1.21	4.27	4.75	5.13	4.59 ^c^	1.22	4.02	4.48	4.99
Cd	100	0.25	1.78	0.06	3.21	0.17	0.24	0.36	0.19 ^a^	1.88	0.12	0.18	0.26	0.20 ^a^	1.51	0.17	0.20	0.26	0.38 ^b^	1.40	0.29	0.39	0.50
Cu *	100	878	1.19	568	1460	775	869	985	884	1.18	786	881	991	880	1.18	765	873	991	869	1.20	782	864	971
Hg	100	0.92	2.28	0.14	10.5	0.48	0.87	1.54	1.00	2.33	0.53	0.93	1.85	0.87	2.14	0.49	0.86	1.43	0.90	2.36	0.47	0.80	1.40
Mn	100	21.1	1.82	9.20	2508	15.5	18.6	23.3	22.6 ^a^	1.75	17.4	20.0	24.5	19.0 ^b^	1.57	13.9	17.3	21.7	21.3 ^a,b^	2.07	15.0	17.8	23.3
Ni	100	3.21	2.35	0.14	90.6	1.91	2.89	5.60	4.43 ^a^	1.87	2.57	5.15	6.45	3.41 ^b^	2.09	1.99	2.82	4.93	2.09 ^c^	2.70	1.16	1.93	3.33
Pb	100	20.8	1.68	7.21	138	14.8	20.4	27.5	24.4 ^a^	1.76	16.8	24.1	33.8	18.1 ^b^	1.49	13.8	17.9	22.5	19.5 ^b^	1.67	13.5	19.0	24.1
Zn	100	3960	1.63	1043	10,211	2765	3953	6024	6142 ^a^	1.29	5351	6233	7298	3420 ^b^	1.54	2447	3351	4962	2703 ^c^	1.38	2252	2799	3228
**% > LD**		**GM**	**GSD**	**MIN**	**MAX**	**P25**	**P50**	**P75**	**GM**	**GSD**	**P25**	**P50**	**P75**	**GM**	**GSD**	**P25**	**P50**	**P75**	**GM**	**GSD**	**P25**	**P50**	**P75**
**Plasma**		**Total (*n* = 246)**							**Micro area I (*n* = 93)**					**Micro area II (*n* = 71)**					**Micro area III (*n* = 82)**				
Al	100	24.2	1.62	5.34	132	17.8	23.4	31.0	29.3 ^a^	1.44	23.5	27.6	34.9	21.2 ^b^	1.71	15.6	19.6	26.3	21.9 ^b^	1.61	16.0	20.7	29.1
As	100	8.33	1.16	5.30	19.8	7.66	8.37	9.00	7.77 ^a^	1.15	7.05	7.81	8.36	8.60 ^b^	1.11	8.17	8.71	9.28	8.76 ^b^	1.17	8.17	8.76	9.38
Cd	99.6	0.03	1.78	0.002	0.53	0.02	0.03	0.04	0.04 ^a^	1.48	0.03	0.04	0.05	0.03 ^b^	2.07	0.02	0.03	0.04	0.03 ^b^	1.70	0.02	0.03	0.04
Cu *	100	969	1.34	484	2301	819	1022	1167	1103 ^a^	1.28	1020	1130	1282	888 ^b^	1.34	685	930	1076	902 ^b^	1.34	755	933	1086
Hg	84.1	0.17	2.36	0.04	2.92	0.11	0.17	0.28	0.16	2.44	0.11	0.17	0.26	0.17	2.30	0.10	0.19	0.29	0.18	2.34	0.12	0.18	0.30
Mg (mg/L)	100	14.2	1.35	5.02	23.8	10.8	13.3	19.1	18.6 ^a^	1.20	17.5	19,5	20.8	14.1 ^b^	1.26	11.3	13.8	17.5	10.6 ^c^	1.14	10.1	10.7	11.4
Mn	100	3.55	1.79	0.85	46.1	2.52	3.27	4.64	4.23 ^a^	1.74	3.00	3.94	5.21	2.52 ^b^	1.59	1.78	2.54	3.28	3.90 ^a^	1.81	2.60	3.26	4.83
Ni	100	1.51	2.09	0.01	46.8	1.34	1.74	2.12	1.51 ^a^	1.53	1.30	1.56	1.93	1.02 ^a^	2.89	0.52	1.36	2.20	2.12 ^b^	1.58	1.71	1.89	2.34
Pb	100	1.46	2.36	0.15	18.1	0.80	1.46	2.43	1.93 ^a^	2.14	1.09	1.71	3.06	0.99 ^b^	2.20	0.55	0.90	1.79	1.48 ^a^	2.46	0.80	1.58	2.49
Zn	100	1022	1.27	628	6389	895	1014	1143	1130 ^a^	1.36	1005	1120	1223	958 ^b^	1.16	849	944	1067	965 ^b^	1.17	881	947	1084

% > LD: % of samples with values above the limit of detection; GM: geometric mean; GSD: geometric standard deviation. MIN: minimum MAX: maximum P: percentile. Al: aluminum; As: arsenic; Cd: cadmium; Cu: copper; Hg: mercury; Mg: magnesium; Mn: manganese; Ni: nickel; Pb: lead; Zn: zinc. * metal with a normal distribution. Superscript letters in GM indicate significant differences (*p*-value ≤ 0.05) in metal concentrations between micro areas (a ≠ b ≠ c).

**Table 5:** Blood metal concentration (mean, µg/L) in the general population and in populations residing near industrial areas in different regions of the world.

Metals	Present Study	Brazil		Europe		Asia		North America		Africa	
		General Population	Near Industries	General Population	Near Industries	General Population	Near Industries	General Population	Near Industries	General Population	Near Industries
**As**	4.8	1.1–4.2	-	1.7	-	2.3	-	0.9	-	-	1.6
**Cd**	0.3	0.1–21.6	-	0.4	0.2–0.8	0.7	1.8–9.1	0.2–0.3	-	-	0.9
**Cu**	878.0	890.0–999.0	-	-	-	784.0	-	900.0	-	-	-
**Hg**	0.9	1.0–1.4	-	0.6–1.4	-	1.9	-	0.7–0.8	-	-	-
**Mn**	21.1	9.6–12.8	-	7.7	12.2	12.4	-	9.6–9.8	-	-	28.5
**Ni**	3.2	0.7–2.1	-	1.3	-	-	-	0.5	-	-	-
**Pb**	20.8	0.5–65.4	-	1.1–18.8	19.7–98.0	17.8	164.8–173.7	8.2–11.0	-	-	-
**Zn**	3960.0	-	-	5805.0	-	5850.0	-	6400.0	-	-	-

References: [3,10,12,13,14,15,16,17,19,20,21,22,23,24,25,54,55].

## Data Availability

Not applicable.

## Supplementary Materials

The following are available online at https://www.mdpi.com/article/10.3390/ijerph182312455/s1, Figure S1: Recruitment protocol of the selected participants, Figure S2: Recruitment protocol of the sex-matched participant, Figure S3: Survey dissemination material, Figure S4: Survey participation invitation card, Table S1: Methods and reference values of hematological and biochemical parameters.
