# Supplementary material for: Biomonitoring of Exposure to Metals in a Population Residing in an Industrial Area in Brazil: A Feasibility Study
Source: Int J Environ Res Public Health. 2021 Nov 26;18(23):12455. doi: 10.3390/ijerph182312455 (PMC8656667; doi:10.3390/ijerph182312455)
Supplement: Supplementary file 1 [file ijerph-18-12455-s001.zip › ijerph-1409326-supplementary.pdf]

Figure S1: Recruitment protocol of the selected participants.

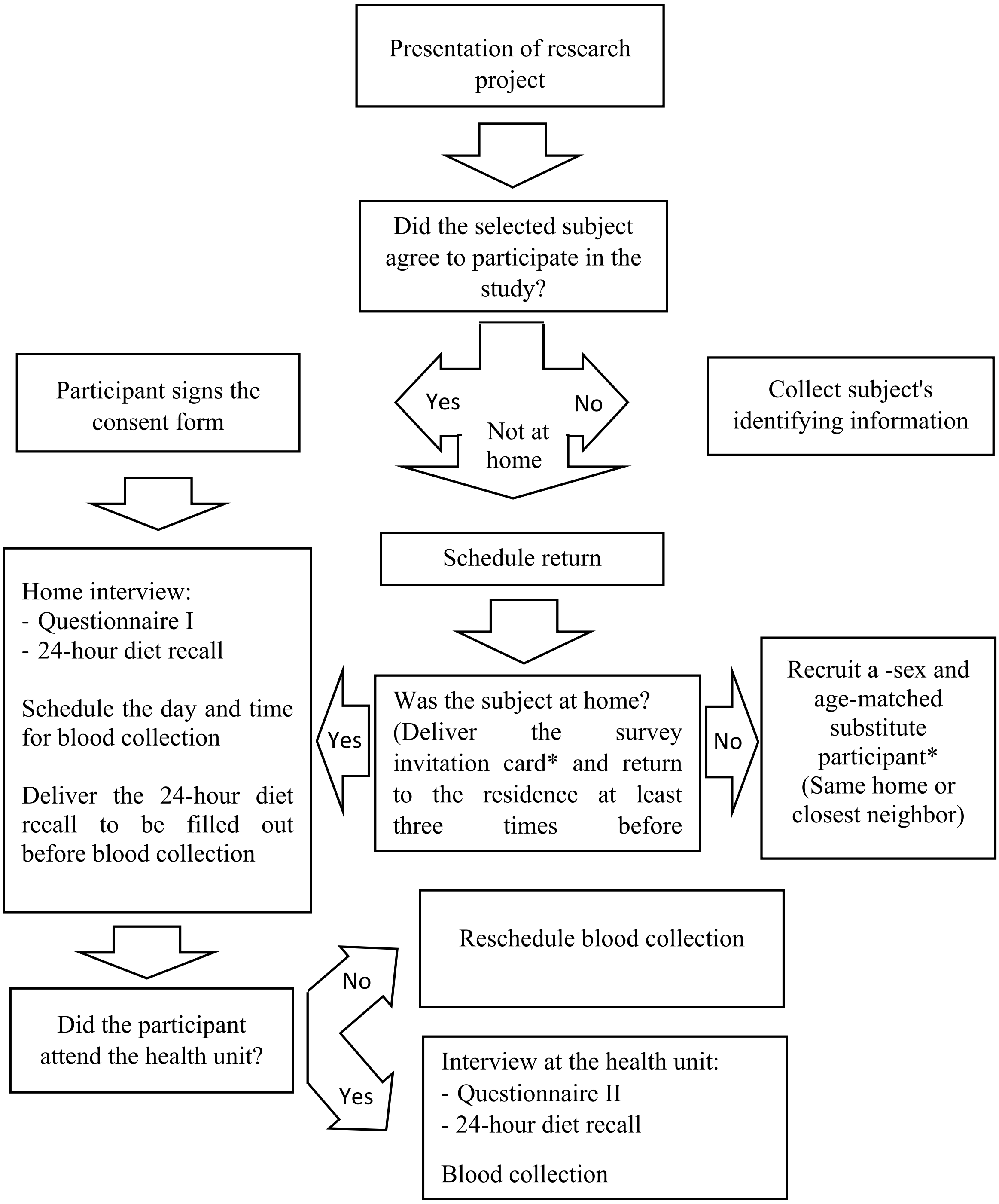

*Age matching and delivery of the research invitation card were strategies established during the pilot study.

Figure S2: Recruitment protocol of the sex-matched participant.

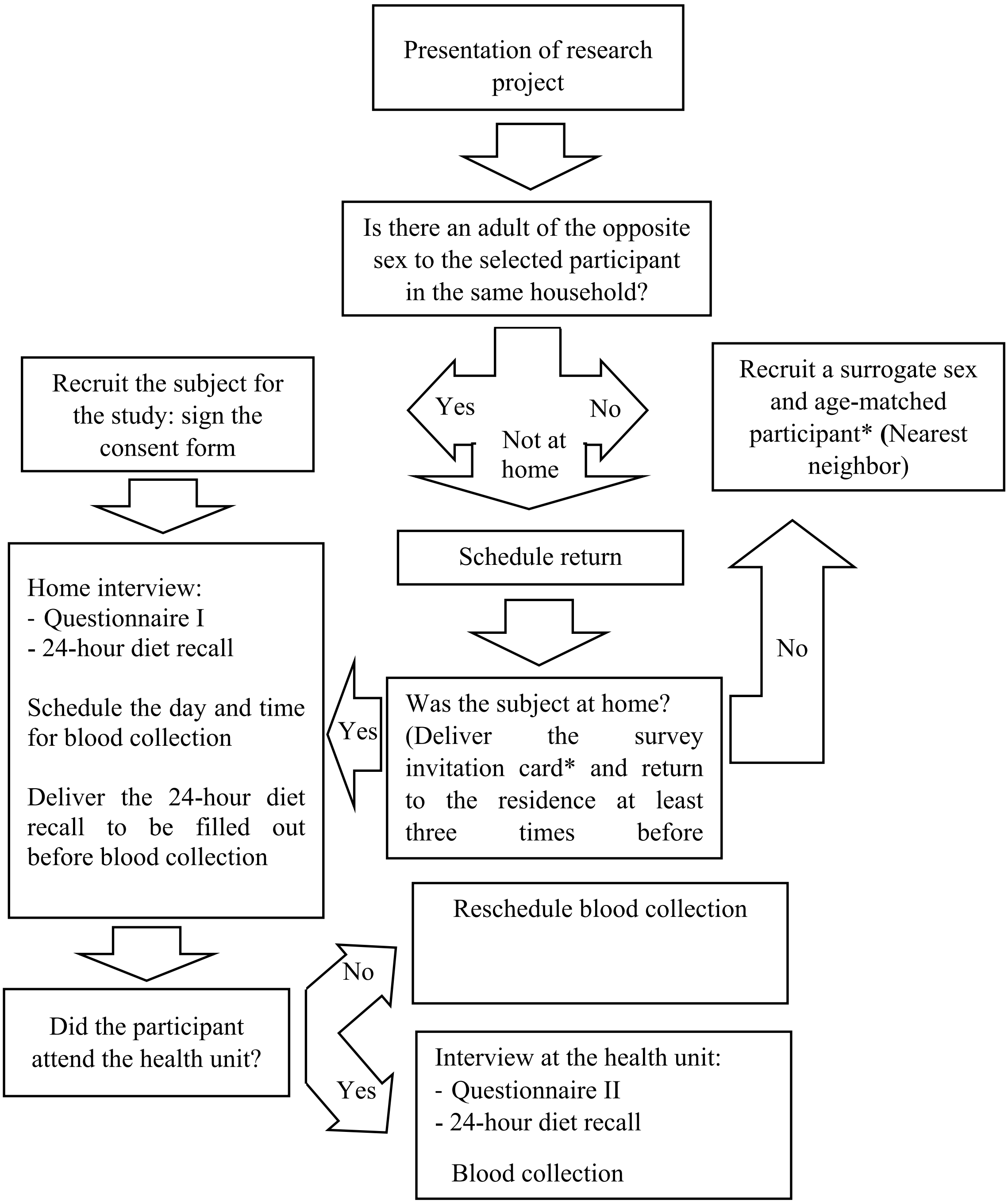

*Age matching and delivery of the research invitation card were strategies established during the pilot study.

Figure S3: Survey dissemination material.

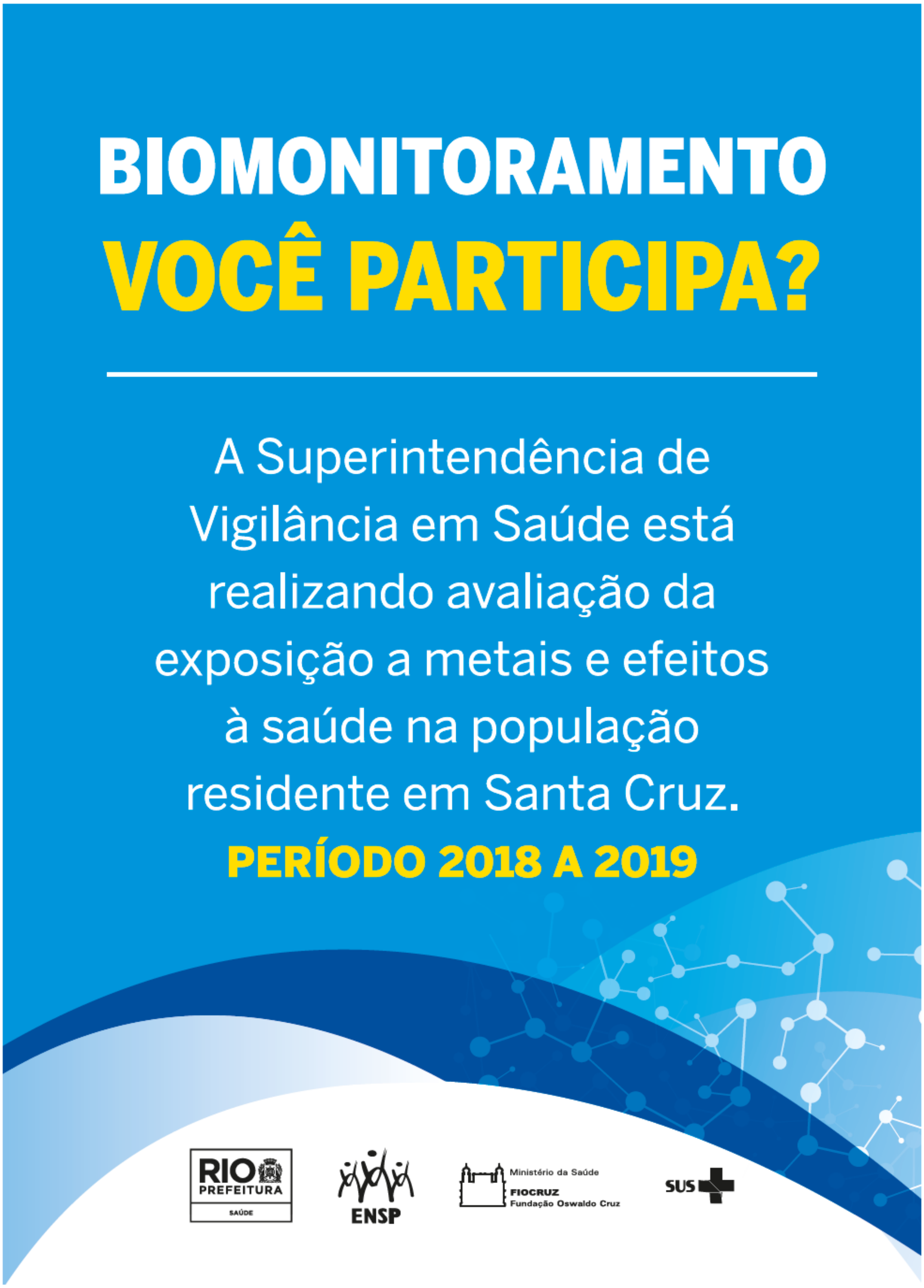

[Content translation: Biomonitoring - Do you participate?
The Health Surveillance Department is investigating exposure to metals and health effects in the population residing in Santa Cruz. Period 2018 to 2019]

Figure S4: Survey participation invitation card.

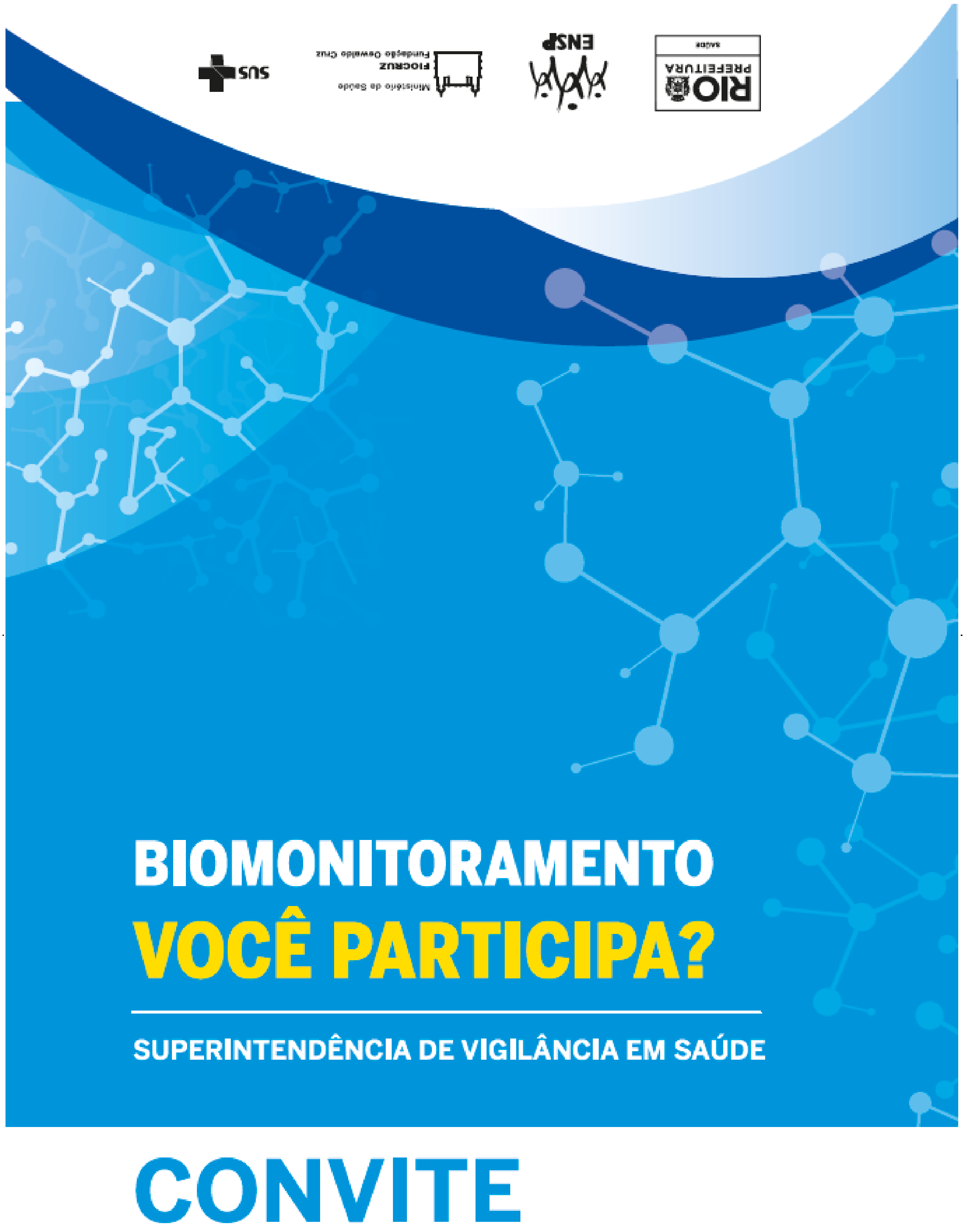

[Content translation: Invitation Card
Biomonitoring - Do you participate?
Health Surveillance Department]

A Superintendência de Vigilância em Saúde está realizando avaliação da exposição a metais e efeitos à saúde na polulação residente em Santa Cruz.

PERÍODO 2018 A 2019

RIO PREFEITURA SAÚDE — ENSP — Ministério da Saúde FIOCRUZ Fundação Oswaldo Cruz — SUS

Prezado(a) Sr(a) ______________________________

Você foi selecionado para avaliação da exposição a metais e efeitos à saúde.

Compareça à ______________________________________
(clínica da família ou CMS)

a partir do dia ____/____, de 2ª a 6ª feira, até as 9h e leve esse documento com você.

Obrigado e até lá!

Rio de Janeiro, _____ / _____ / _____

____
Agente de Vigilância em Saúde

[Content translation: The Health Surveillance Department is investigating exposure to metals and health effects in the population residing in Santa Cruz. Period 2018 to 2019. Dear Mr./Mrs. *(participant name)*. You have been selected to participate in the metal exposure and health effects assessment study. Attend the *(health unit name)* from day *(date of appearance)* from Monday to Friday, until 9 am and take this document with you. Thanks, and see you there!]

Table S1: Methods and reference values of hematological and biochemical parameters.

Parameter	Method	Reference value
Blood count	Automation	
Red blood cells		men= 4.4-5.9; woman= 4.0-5.4 10^6/μL
Hemoglobin		men= 13.0-18.0; woman=12.0-16.0 g/dL
Hematocrit		men= 40-52; woman= 35-47%
MCV		80-100 fL
MCH		27-32 pg
CMCH		32-37 g/dL
RDW		≤15%
White blood cells		100% or 3.5-11 10^3/μL
Neutrophils		50-70% or 2.5-7 10^3/μL
Eosinophils		1-6% or 0.1-0.6 10^3/μL
Basophils		0-2% or 0-0.2 10^3/μL
Lymphocytes		20-30% or 1-3 10^3/μL
Monocytes		2-12% or 0.1-0.8 10^3/μL
Platelets		150-450 10^3/μL
Total cholesterol	Automation by CHOD-POD	18-19 years: desirable ≤169; borderline= 170-199; high >199<br>>19 years: desirable ≤199; borderline= 200-239; high >239 mg/Dl
Triglycerides	Automation by GPO-POD	18-19 years: desirable ≤130<br>>19 years: normal ≤149; borderline= 150-199; high= 199-499; very high >499 mg/dL
Creatinine	Automation by kinetic alkaline picrate (Jaffe) reaction	men= 0.7-1.3; woman= 0.5-1.1 mg/dL
Urea	Automation - UV enzymatic - urease/glutamate	15-50 mg/dL
AST	Automation - UV optimized by IFCC	men= 15-40; woman= 13-35 U/L
ALT	Automation - UV optimized by IFCC	men= 10-40; woman= 7-35 U/L
Alkaline phosphatase	Automation - Kinetic optimized by IFCC	18-19 years: men ≤155; woman ≤150<br>>19 years= 25-100 U/L
TSH	Chemiluminescence	18-60 years= 0.4-4.3 μUmI/mL<br>61-79 years= 0.4-5.8 μUmI/mL<br>>79 years= 0.4-6.7 μUmI/mL
FT4	Chemiluminescence	0.70-1.80 ng/dL
TT3	Chemiluminescence	70-220 ng/dL

MCV: mean cell volume; MCH: mean cell hemoglobin; CMCH: concentration of mean cell hemoglobin; RWD: red cell distribution width; AST: aspartate aminotransferase; ALT: alanine aminotransferase; TSH: thyroid-stimulating hormone; TT3: total triiodothyronine; FT4: serum levels of free thyroxine. UV: ultraviolet. IFCC: International Federation of Clinical Chemistry. CHOD-POD: cholesterol oxidase and peroxidase. GPO-POD: glycerol phosphate oxidase-peroxidase.
